# Supplementary material for: Impact of Sidestream Pre-Treatment on Ammonia Recovery by Membrane Contactors: Experimental and Economic Evaluation
Source: Membranes (Basel). 2022 Dec 10;12(12):1251. doi: 10.3390/membranes12121251 (PMC9787290; doi:10.3390/membranes12121251)
Supplement: Supplementary file 1 [file membranes-12-01251-s001.zip › Supplementary Information Impact of sidestream pre-treatment on ammonia recovery by membrane contactors Experimental and economic evaluation TEMPLATE MDPI.pdf]

Table S1. Initial conditions for coagulant selection

Coagulant	*Dosage (mg/L)*	*Mixing time (min)*	*Mixing speed (rpm)*	*Settling time (min)*
$FeCl_3$				
$Al_2(SO_4)_3$	50	5	200	30
Derypol HT20				

Table S2. Flocculation parameters

Flocculation Parameters	
Mixing time	30 min
Mixing speed	30 rpm
Resting time	30 min
Dosage	10, 25, 30, 40 and 50 mg
Flocculant	Fe_3O_4 + SiO_2 (30-70%)

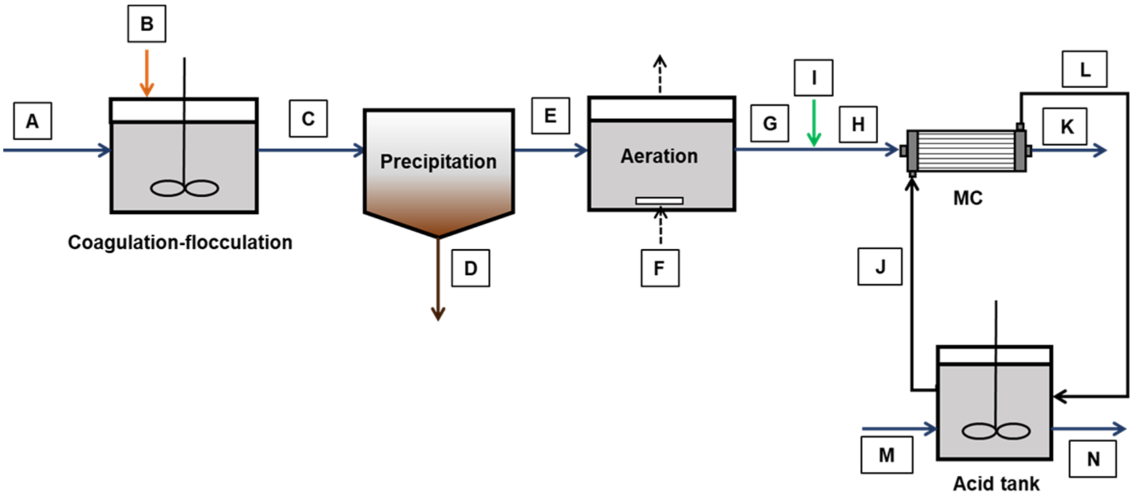

Figure S1. Schematic representation of the nitrogen recovery scheme.

Table S3. Main flow data for the nitrogen recovery scenario under study.

(A) **Anaerobic digester centrate**	
Flow rate (m^3/day)	150
TSS (g/L)	0.24
TAN (g N/L)	0.71
pH (-)	8.1
**(B) $Al_2(SO_4)_3$ solution**	
Flow rate (m^3/day)	9
$Al_2(SO_4)_3$ (mg/L)	500
**(C) Coagulation-flocculation effluent**	
Flow rate (m^3/day)	159
TSS (g/L)	0.22

TAN (g N/L)	0.67
pH (-)	8.1
**(D) Solid fraction precipitator**	
Flow rate (m^3/day)	8
TSS (g/L)	3.98
**(E) Liquid fraction precipitator**	
Flow rate (m^3/day)	151
TAN (g N/L)	0.67
pH (-)	7.9
**(F) Air**	
Flow rate (Nm^3/day)	2288
**(G) Effluent aeration tank**	
Flow rate (m^3/day)	151
TAN (g N/L)	0.67
pH (-)	8.9
**(I) NaOH solution**	
Flow rate (m^3/day)	0.3
NaOH (mol/L)	1
**(H) Feeding solution $MC_{t=0}$**	
Flow rate (m^3/day)	151.3
TAN (g N/L)	0.67
pH (-)	10.3
**(K) Feeding solution $MC_{t=F}$**	
Flow rate (m^3/day)	151.3
TAN (g N/L)	0.24
pH (-)	8.42
**(M) HNO_3 solution**	
Volume/cycle (m^3/cycle)	0.52
Number of cycles (cycles/day)	22
HNO_3 (mol/L)	0.4
pH (-)	0.4
**(N) NH_4NO_3 solution**	
Volume/cycle (m^3/cycle)	0.52
Number of cycles (cycles/day)	22
NH_4NO_3 (mol/L)	0.4
pH (-)	6

Table S4. Main design parameters used for the economic evaluation.

	Parameter	Value	Source
**Coagulation-flocculation**	Specific $Al_2(SO_4)_3$ consumption ($g\ Al_2(SO_4)_3\ /L_{centrate}$)	0.19	Lab-scale data
	Retention time (h)	0.1	Lab-scale data
	Mixer revolutions (rpm)	100	Lab-scale data
**Precipitation**	Retention time (h)	0.5	Lab-scale data
	Q_0/Q_E (%)	95	Lab-scale data
**Aeration**	Retention time (h)	1	Lab-scale data

	Specific air consumption (NL/h/L_{tank})	364	Lab-scale data
**Membrane contactor**	K_m (m/s)	1.04×10^{-5}	Lab-scale data
	TAN recovery (%)	64	Lab-scale data
	Flow rate trapping solution:Flow rate feed solution	1:1	Lab-scale data
	Specific NaOH consumption (mol NaOH/mol $TAN_{recovered}$)	0.067	Lab-scale data
	$pH_{t=0}$ HNO_3 trapping solution (-)	0.4	Lab-scale data
	$pH_{t=F}$ HNO_3 trapping solution (-)	6	[1]

Table S5. Main economic parameters used for the economic evaluation.

Parameter	Value	Source
Tank cost (€/m^3)	220	[2]
Settler cost (€/m^3)	100	[3]
Pump cost (€/m^3/h)	12.1	[2]
Stirrer cost (€/m^3_{Tank})	27.8	[4]
Blower cost (€/Nm^3/h)	4.15	[2]
Membrane cost (€/m^2)	49	[3]
$Al_2(SO_4)_3$ cost (€/kg)	0.16	[5]
NaOH cost (€/kg)	0.62	[6]
HNO_3 cost (€/kg)	0.38	[7]
Electricity cost (€/kWh)	0.1445	[8]
Sludge disposal cost (€/t TS)	373	[9]
Lifetime membrane (years)	10	[3]
Lifetime auxiliary equipment (years)	10	[3]
NH_4NO_3 price (€/kg)	0.43	[10]
Energy consumption mainstream N removal (kWh/kg N)	2.38	[11]

Table S7. Experiments set of Design Expert 11 software.

Run	*M. Time (min)*	*M. Speed (rpm)*	*S. Time (min)*	*Run*	*M. Time (min)*	*M. Speed (rpm)*	*S. time (min)*
1	25	100	45	***10***	25	250	15
2	15	175	30	***11***	5	100	45
3	5	250	15	***12***	5	250	45
4	15	175	30	***13***	15	175	37,5
5	25	250	45	***14***	10	175	30
6	25	100	15	***15***	5	100	15
7	15	137,5	30	***16***	15	175	30
8	15	175	22,5	***17***	15	212,5	30
9	20	175	30				

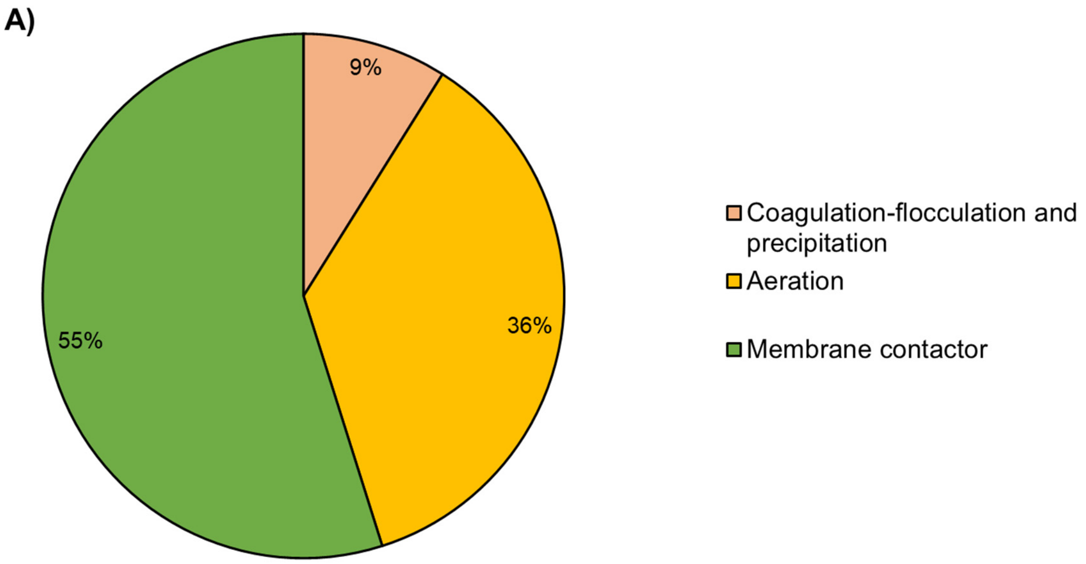

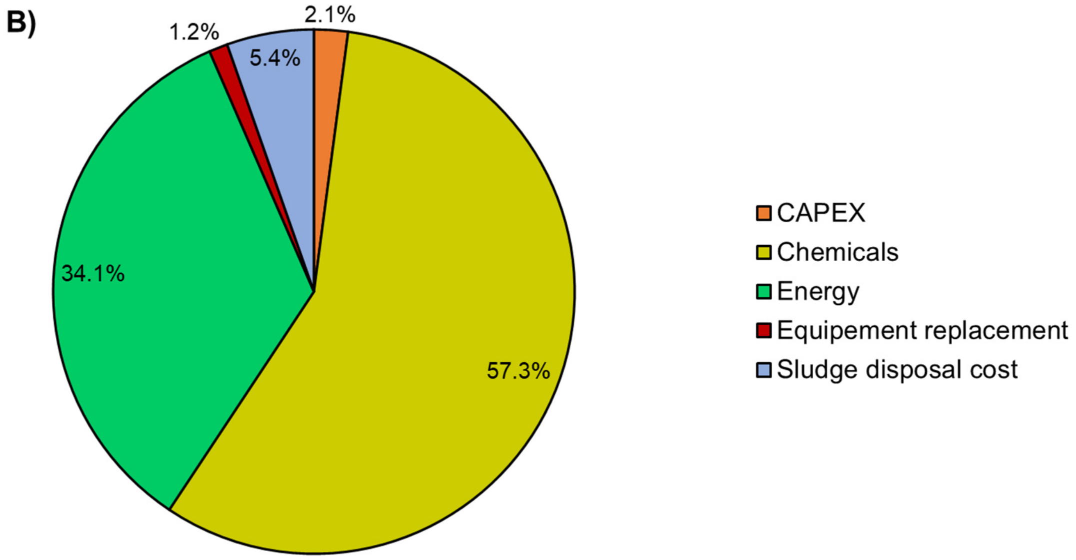

Figure S2. Gross cost contribution of the nitrogen recovery scenario under study for: (A) the different processes and (B) for the different capital and operating costs.

1. Richter, L.; Wichern, M.; Grömping, M.; Robecke, U.; Haberkamp, J. Ammonium Recovery from Process Water of Digested Sludge Dewatering by Membrane Contactors. *Water Pract. Technol.* **2020**, *15*, 84–91, doi:10.2166/wpt.2020.002.
2. Verrecht, B.; Maere, T.; Nopens, I.; Brepols, C.; Judd, S. The Cost of a Large-Scale Hollow Fibre MBR. *Water Res.* **2010**, *44*, 5274–5283, doi:10.1016/j.watres.2010.06.054.
3. Noriega-Hevia, G.; Serralta, J.; Seco, A.; Ferrer, J. Economic Analysis of the Scale-up and Implantation of a Hollow Fibre Membrane Contactor Plant for Nitrogen Recovery in a Full-Scale Wastewater Treatment Plant. *Sep. Purif. Technol.* **2021**, *275*, 119128, doi:10.1016/j.seppur.2021.119128.
4. Vinardell, S.; Astals, S.; Mata-Alvarez, J.; Dosta, J. Techno-Economic Analysis of Combining Forward Osmosis-Reverse Osmosis and Anaerobic Membrane Bioreactor Technologies for Municipal Wastewater Treatment and Water Production. *Bioresour. Technol.* **2020**, *297*, 122395, doi:10.1016/j.biortech.2019.122395.
5. Vu, H.P.; Nguyen, L.N.; Lesage, G.; Nghiem, L.D. Synergistic Effect of Dual Flocculation between Inorganic Salts and Chitosan on Harvesting Microalgae Chlorella Vulgaris. *Environ. Technol. Innov.* **2020**, *17*, 100622, doi:10.1016/j.eti.2020.100622.
6. Bouzas, A.; Martí, N.; Grau, S.; Barat, R.; Mangin, D.; Pastor, L. Implementation of a Global P-Recovery System in Urban Wastewater Treatment Plants. *J. Clean. Prod.* **2019**, *227*, 130–140, doi:10.1016/j.jclepro.2019.04.126.
7. Das, S.; Gaustad, G.; Sekar, A.; Williams, E. Techno-Economic Analysis of Supercritical Extraction of Rare Earth Elements from Coal Ash. *J. Clean. Prod.* **2018**, *189*, 539–551, doi:10.1016/j.jclepro.2018.03.252.
8. Eurostat Electricity Price Statistics. **2021**.
9. Foladori, P.; Andreottola, G.; Ziglio, G. *Sludge Reduction Technologies in Wastewater Treatment Plants*; IWA Publishing, 2015; ISBN 9781780401706.
10. Ministerio de Agricultura Pesca y Alimentación Índices y Precios Pagados Agrarios 2022.
11. Horstmeyer, N.; Weißbach, M.; Koch, K.; Drewes, J.E. A Novel Concept to Integrate Energy Recovery into Potable Water Reuse Treatment Schemes. *J. Water Reuse Desalin.* **2018**, *8*, 455–467, doi:10.2166/wrd.2017.051.
